# A comparative study of X-ray tomographic microscopy on shales at different synchrotron facilities: ALS, APS and SLS

**DOI:** 10.1107/S0909049512044354

**Published:** 2012-11-22

**Authors:** Waruntorn Kanitpanyacharoen, Dilworth Y. Parkinson, Francesco De Carlo, Federica Marone, Marco Stampanoni, Rajmund Mokso, Alastair MacDowell, Hans-Rudolf Wenk

**Affiliations:** a Department of Earth and Planetary Science, University of California, Berkeley, CA 94720, USA; b Advanced Light Source, Lawrence Berkeley National Laboratory, Berkeley, CA 94720, USA; c Advanced Photon Source, Argonne National Laboratory, IL 60439, USA; d Swiss Light Source, Paul Scherrer Institute, CH-5232 Villigen, Switzerland; e Institute for Biomedical Engineering, University and ETH Zürich, CH-8092 Zürich, Switzerland

**Keywords:** X-ray tomographic microscopy, shale, porosity, microstructure

## Abstract

Synchrotron radiation X-ray tomographic microscopy (SRXTM) was used to characterize the three-dimensional microstructure, geometry and distribution of different phases in two shale samples obtained from the North Sea (sample N1) and the Upper Barnett Formation in Texas (sample B1). Shale is a challenging material because of its multiphase composition, small grain size, low but significant amount of porosity, as well as strong shape- and lattice-preferred orientation. The goals of this round-robin project were to (i) characterize microstructures and porosity on the micrometer scale, (ii) compare results measured at three synchrotron facilities, and (iii) identify optimal experimental conditions of high-resolution SRXTM for fine-grained materials. SRXTM data of these shales were acquired under similar conditions at the Advanced Light Source (ALS) of Lawrence Berkeley National Laboratory, USA, the Advanced Photon Source (APS) of Argonne National Laboratory, USA, and the Swiss Light Source (SLS) of the Paul Scherrer Institut, Switzerland. The data reconstruction of all datasets was handled under the same procedures in order to compare the data quality and determine phase proportions and microstructures. With a 10× objective lens the spatial resolution is approximately 2 µm. The sharpness of phase boundaries in the reconstructed data collected from the APS and SLS was comparable and slightly more refined than in the data obtained from the ALS. Important internal features, such as pyrite (high-absorbing), and low-density features, including pores, fractures and organic matter or kerogen (low-absorbing), were adequately segmented on the same basis. The average volume fractions of low-density features for sample N1 and B1 were estimated at 6.3 (6)% and 4.5 (4)%, while those of pyrite were calculated to be 5.6 (6)% and 2.0 (3)%, respectively. The discrepancy of data quality and volume fractions were mainly due to different types of optical instruments and varying technical set-ups at the ALS, APS and SLS.

## Abstract 2

The 3D microstructure of shales is important to assess elastic anisotropic characteristics. In this study, microporosity and mineral components in two shale samples were investigated with X-ray tomographic microscopy at three synchrotron facilities: ALS, APS and SLS, and excellent agreement was observed.

## 1. Introduction   

X-ray absorption tomographic microscopy is a non-destructive, high-resolution and three-dimensional (3D) imaging method, which is based on different linear attenuation coefficients of constituent phases (Beer–Lambert’s law). The technique has long been used to characterize microstructures of a wide variety of materials such as biomedical specimens (*e.g.* Agatston *et al.*, 1990 ▶), engineering materials (*e.g.* Beckmann *et al.*, 2007 ▶; Meirer *et al.*, 2011 ▶), concretes (*e.g.* Monteiro *et al.*, 2009 ▶), fossils (*e.g.* Gai *et al.*, 2011 ▶) and food products (*e.g.* Müller *et al.*, 2011 ▶). Recently, tomography has been performed with synchrotron X-ray sources, which provide a brilliant and intense X-ray beam. The high X-ray brilliance allows us to carry out experiments with *ad hoc* tuned monochromatic radiation, resulting in low-noise data with optimal contrast. Advances in technology further enable synchrotron radiation X-ray tomographic microscopy (SRXTM) experiments to be performed *in situ* to study different properties; for instance, the localized corrosion of aluminium (Connolly *et al.*, 2006 ▶), the water transport paths in gas diffusion layers of fuel cells (Markötter *et al.*, 2011 ▶), and the interface between iron alloy droplet and silicate melt under high pressure and temperature (Terasaki *et al.*, 2009 ▶).

With its growing importance in the geological field, SRXTM has been applied to various geomaterials such as sandstone (Lindquist *et al.*, 2000 ▶), mylonite (Fusseis *et al.*, 2009 ▶), peridotite (Zhu *et al.*, 2011 ▶), volcanic rock (Voltolini *et al.*, 2011 ▶), meteorites (Friedrich *et al.*, 2008 ▶), gypsum (Fusseis *et al.*, 2012 ▶) and shale (Lenoir *et al.*, 2007 ▶; Kanitpanyacharoen *et al.*, 2011 ▶, 2012 ▶). Shales are of interest owing to the low porosity and permeability, which allow them to serve as cap rocks for hydrocarbon reservoirs (Best & Katsube, 1995 ▶), repository sites for nuclear wastes (Mallants *et al.*, 2001 ▶; Bossart & Thury, 2007 ▶), and storehouses for carbon sequestration (Chadwick *et al.*, 2004 ▶; Busch *et al.*, 2008 ▶). During seismic surveys of shales, elastic waves travel significantly faster along the bedding plane than the bedding normal direction. This phenomenon is known as elastic anisotropy, mainly caused by the shape and lattice-preferred orientation of constituent phases. While mineral lattice orientation distribution can be derived from synchrotron X-ray diffraction experiments (*e.g.* Wenk *et al.*, 2010 ▶), the 3D shape orientation distribution is difficult to quantify owing to their complex microstructures, small grain sizes and multiphase composition. Besides, the availability of software and their ability to adequately segment components of interests are challenging problems for high-spatial-resolution investigations.

In this study, two shales were analyzed to determine 3D internal features, to explore resolution limitations of three SRXTM beamlines of different third-generation synchrotron sources, and to develop satisfactory procedures for data quantification. SRXTM data were collected at beamline 8.3.2 at the Advanced Light Source (ALS) of Lawrence Berkeley National Laboratory, USA, beamline 2-BM at the Advanced Photon Source (APS) of Argonne National Laboratory, USA, and beamline TOMCAT at the Swiss Light Source (SLS) of the Paul Scherrer Institut, Switzerland. Several other beamlines such as BL6-2 at Stanford Synchrotron Radiation Laboratory in USA, ID15 and ID19 at the European Synchrotron Radiation Facility in France, BL20XU at SPring-8 in Japan, BL6.1R at Elettra Synchrotron in Italy, and P05 IBL at Deutsches Elektronen-Synchrotron in Germany are also capable of performing high-resolution SRXTM but here we concentrate on the three facilities mentioned above. The data were collected with the same parameters to compare the quality of the reconstructed data. All datasets were reconstructed and quantified at the ALS.

## 2. Samples   

Two well characterized shales were selected for this study. The first sample is a Kimmeridge-aged shale from a borehole at 3750 m in the North Sea of England and is referred to as N1. Previous studies suggest that N1 has a porosity of 2.5% and is composed of illite-smectite-mica (35 wt%), quartz (30 wt%), kaolinite (22 wt%), pyrite (4 wt%), feldspar (7%) and chlorite (2 wt%) (Hornby, 1998 ▶). The lattice-preferred orientation was quantified, suggesting strong alignment of (001) clay platelets parallel to the bedding plane with maximum concentrations of six multiples of random distribution (m.r.d) for kaolinite, 4 m.r.d. for illite-mica, and 2 m.r.d. for illite-smectite (Wenk *et al.*, 2010 ▶).

The second sample is a shale from the Upper Barnett Formation of Late Mississippian age of Fort Worth Basin in Texas from a borehole at 2167 m depth, and is referred to as B1 (Day-Stirrat *et al.*, 2008 ▶). A large amount of fine-grained illite-smectite (23.7 wt%) and illite-mica (17.9 wt%) is present in sample B1, along with coarse-grained quartz (44.0 wt%), calcite (6.8 wt%), feldspars (3.1 wt%), dolomite (2.6 wt%) and pyrite (1.5 wt%). The degree of preferred orientation ranges from 2 m.r.d. (illite-smectite) to 7 m.r.d. (illite-mica) (Day-Stirrat *et al.*, 2008 ▶).

Both samples were first cut into small rectangular prisms (1 mm × 1 mm × 5 mm) with the aid of kerosene as a cooling agent. The small prisms were glued on a glass slide and polished with a file tool into small cylinders (1 mm diameter × 5 mm length) for the SRXTM experiments.

## 3. Methods   

### 3.1. Data acquisition   

**Figure 1:**
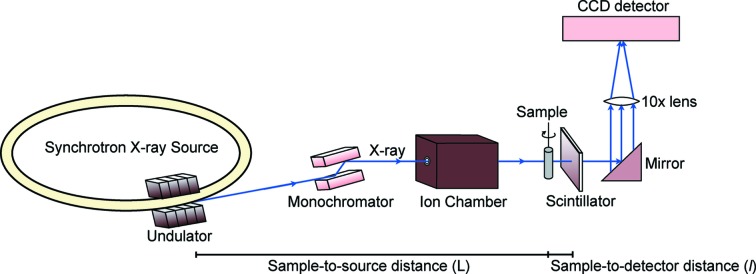
Schematic diagram of the SRXTM experiment.

A typical SRXTM experimental set-up at a synchrotron is illustrated in Fig. 1 ▶. Each synchrotron facility, however, has different technical configurations and specifications for equipment (Table 1 ▶). More details of each beamline are described elsewhere (Parkinson, 2012 ▶; Wang *et al.*, 2001 ▶; Stampanoni *et al.*, 2006 ▶). First, several bright- and dark-field images were collected for X-ray fluctuation correction and background normalization. Bright-field images were collected with X-ray beam illumination but without the sample in the field of view (FOV) whereas dark-field images were acquired for detector background without the X-ray beam. The correction method is briefly described in §3.2.

**Figure 2:**
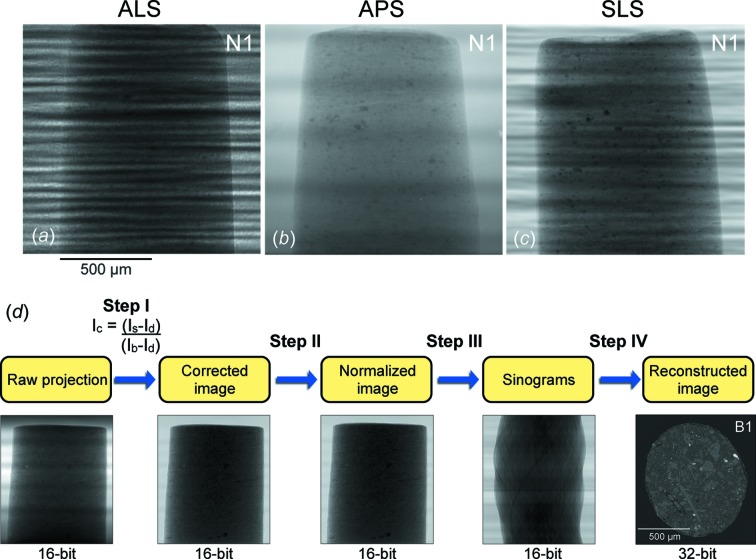
(*a*)–(*c*) Single raw projection images of sample N1 collected at each synchrotron facility, and (*d*) a workflow of data reconstruction with sample B1 as an example.

During the experiment the cylindrical sample was mounted on a rotational stage with its long axis vertical and centered in the FOV. The sample was rotated in 0.120° incremental steps for a total of 180° during a continuous rotation with a monochromatic X-ray energy of 18 keV, corresponding to a wavelength of 0.689 Å. The exposure time was different for the beamlines (Table 1 ▶). The transmitted X-ray intensity was absorbed by a thin scintillator screen, which converts X-rays to a certain wavelength of the visible light, depending on scintillator material. The visible light was further projected onto a CCD detector through a 10× objective lens. Each raw projection represents a two-dimensional X-ray attenuation map, which was used to reconstruct a 3D data volume. Raw projections of N1 are similar to those of sample B1, thus only examples of sample N1 from each facility are displayed in Figs. 2(*a*)–2(*c*) ▶. The raw projections from the ALS and the SLS were written as Tagged Image Files (TIFs) while those from the APS were created in the Hierarchical Data Format (HDF) (Wang *et al.*, 2001 ▶). The HDF images were converted to TIF format by a code written in Matlab for consistency in data reconstruction.

### 3.2. Data reconstruction   

In general, each beamline uses different software for SRXTM data reconstruction. *Octopus* software (Dierick *et al.*, 2004 ▶), which relies on a filtered back-projection algorithm, is normally employed at beamline 8.3.2 of the ALS. Beamline 2-BM of the APS and beamline TOMCAT of the SLS use their in-house-developed applications with a code based on the Gridrec algorithm and fast Fourier transforms (Dowd *et al.*, 1999 ▶; De Carlo & Tieman, 2004 ▶; Hintermüller *et al.*, 2010 ▶). Each software has its own advantages and disadvantages but the analysis of software is not the purpose of this study. The data reconstruction was performed at the ALS, thus only *Octopus* software was used to establish a reasonable comparison of data quality.

Reconstruction involves multiple steps of data processing as shown in Fig. 2(*d*) ▶. In step I, raw projection images were corrected with background images (bright- and dark-field) to remove the smearing effect on sharp details (or artifacts), resulting from X-ray beam fluctuation and defects in monochromator, scintillator, objective lens and detector. The following method (Wang *et al.*, 2001 ▶) was used to correct the images: *I*_c_ = [(*I*_s_ − *I*_d_)/(*I*_b_ − *I*_d_)], where *I*_c_ is the corrected image, *I*_b_ is the bright-field image, *I*_d_ is the dark-field image and *I*_s_ is the raw projection of sample. The corrected images were normalized in step II by choosing a region of the images which contains no sample, and finding the average value in that region to produce the same grayscale levels for all images in the dataset. In step III the normalized data were then rearranged into a sinogram, which contains information of all projection angles of a projection horizontal line. A few concentric rings can be observed in the sinograms owing to defective pixels in the detector that are present at the same coordinates in all projections (Dierick *et al.*, 2004 ▶). These artifacts were thus removed by a minimal level of median filter (level 1). The ring filter first determined the mean of the pixel value in each column of the sinogram and compared it with its eight neighboring pixels. The pixels in the column that have a higher deviation than the chosen level were then replaced by multiplying with a correction factor (Dierick *et al.*, 2004 ▶). After obtaining filtered sinograms, the center of the sample’s rotation was calculated from the projections at 0° and 180° in step IV. The data were further reconstructed based on the filtered back-projection algorithm (Dierick *et al.*, 2004 ▶) and represented in 32-bit TIF format (2048 × 2048 pixels). The 32-bit TIF uses floating-point numbers to represent a wide range of grayscale values (2^32^ shades) in the sample.

**Figure 3:**
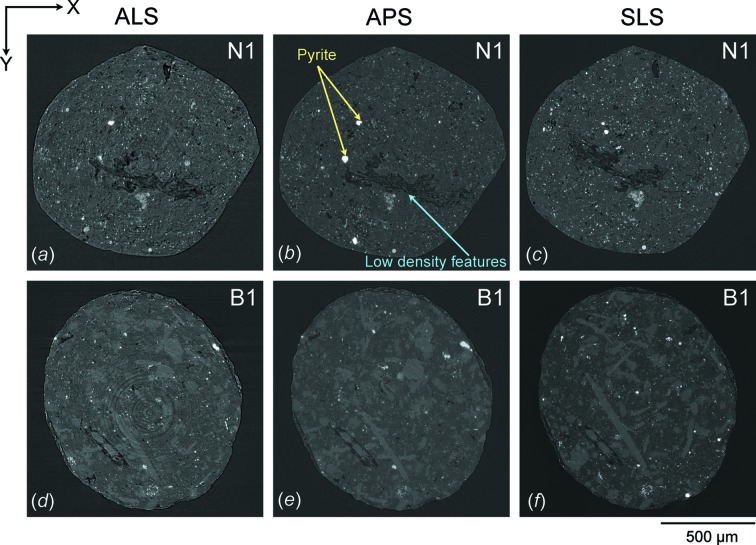
Reconstructed images of (*a*)–(*c*) sample N1 and (*d*)–(*f*) sample B1 obtained from each synchrotron facility.

**Figure 4:**
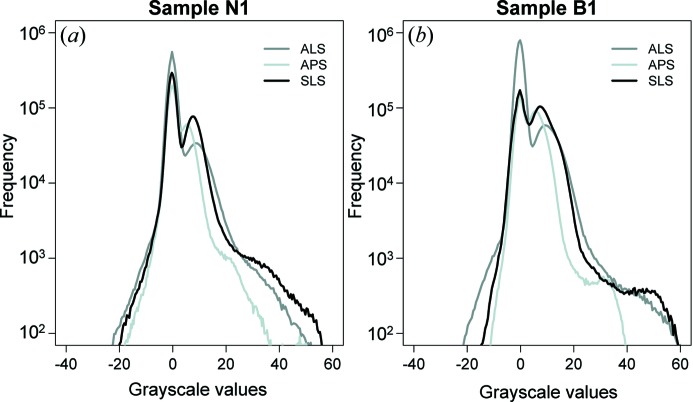
Histogram plots of grayscale values on a logarithmic scale of (*a*) sample N1 and (*b*) sample B1 obtained from each facility. Note that the grayscales were extracted from the same cropped area in Figs. 5 ▶ and 6 ▶.

The same procedures were repeated for all datasets. A similar slice of both sample N1 [Figs. 3(*a*)–3*c* ▶] and B1 [Figs. 3(*d*)–3(*f*)] were identified for comparison. Small variations in sample tilts, especially in the SLS measurement, contribute to slightly shifted views. The histograms of grayscale value extracted from all measurements were plotted on a logarithmic scale and are shown in Fig. 4 ▶. Overall, the grayscale values of all datasets display comparable ranges in the histogram. The ALS data have a relatively wider range of grayscales (Table 2 ▶: −30.37 to 72.55 for N1 and −23.67 to 68.97 for B1) and a more pronounced negative tail in the histogram (Fig. 4 ▶). The histogram of APS data contains the smallest ranges (Table 2 ▶: −12.58 to 54.32 for N1 and −10.61 to 42.64) and falls within the ALS and SLS gray values. Note that grayscale of absorption = −ln(%Transmission) = −ln[(*I*_s_ − *I*_d_)/(*I*_b_ − *I*_d_)]. Negative grayscale in the final reconstructed image corresponds to a %Transmission of greater than 100%, which is when a pixel has a higher value for *I*_s_ than for *I*_b_. This can occur due to noise and fluctuations in the incident X-ray beam, or due to phase-contrast artifacts. The phase-contrast contribution likely explains the more pronounced negative tail in the histogram for the ALS, which has greater phase-contrast contributions.

### 3.3. Data quantification   

Several software packages for 3D tomographic data analysis are available (*e.g.* Lindquist, 2002 ▶; Ketcham, 2005 ▶; Modular Algorithms for Volume Images, 2005 ▶; Brun *et al.*, 2010 ▶; Brabant *et al.*, 2011 ▶, *etc.*) but the ‘Quantification’ tool in *Avizo Fire* software (version 6) (Visualization Sciences Group; Massachusetts, USA) was used for segmentation in all datasets.

**Figure 5:**
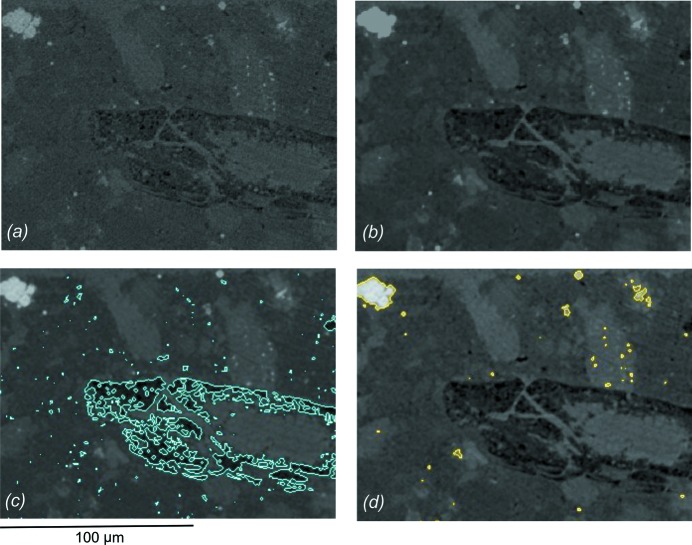
Images in the *XY*-plane of sample B1 obtained from the APS display an axial reconstructed slice (*a*) before and (*b*) after applying a 3D median filter, as well as the thresholding boundary of (*c*) low-density features and (*d*) pyrite.

**Figure 6:**
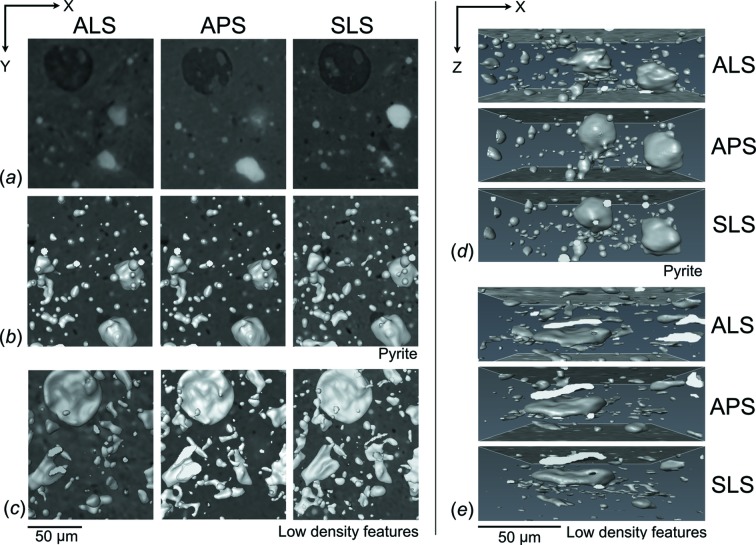
Images in the *XY*-plane show (*a*) axial slices through the cropped reconstructed volume of sample N1 after applying a 3D median filter, (*b*) the segmentation of pyrite, and (*c*) low-density features in 3D. Alternate views in the *XZ*-plane of the geometry and 3D distribution of (*d*) pyrite and (*e*) low-density features are also displayed.

Each dataset was input with its corresponding pixel size (0.88 µm for the ALS, 0.72 µm for the APS, and 0.74 µm for the SLS) and processed with a 3D median filter. This filter reduces noise by replacing the grayscale value of each voxel with a median of its neighborhood within 3 × 3 × 3 voxel window. Figs. 5(*a*) and 5(*b*) ▶ illustrate the difference between before and after applying the median filter to the reconstructed slice of sample B1. A small volume of interest (VOI) of 250 µm × 580 µm × 50 µm was selected from sample B1 to emphasize distinctive features (Fig. 6 ▶). Different components in the filtered data can then be segmented by the thresholding method implemented in *Avizo*. The threshold values separate the image into background and foreground (binary) by assigning a label to every voxel and effectively distinguishing between low- and high-absorbing phases.

**Figure 7:**
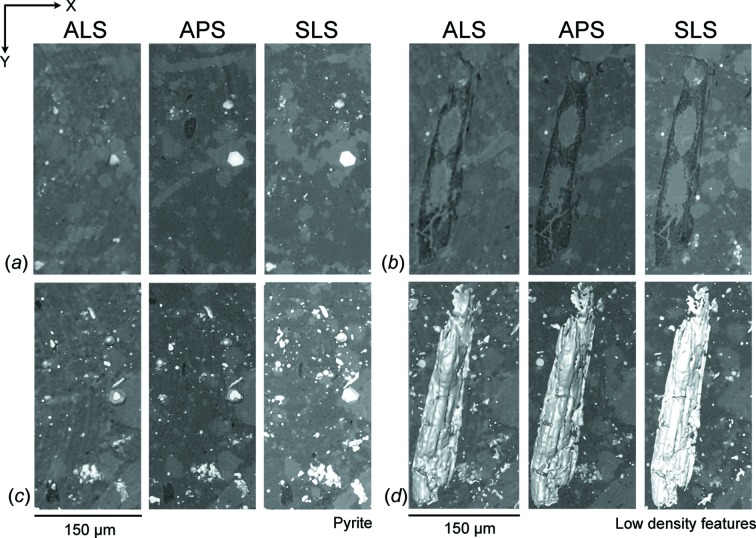
Images in the *XY*-plane show axial slices through the cropped reconstructed volume of sample B1 after applying a 3D median filter in (*a*)–(*b*), the segmentation of pyrite (*c*), and low-density features (*d*) in 3D.

The highly absorbing particles (white) are pyrite (Figs. 5 ▶, 6 ▶ and 7 ▶) while intermediate shades are a combination of clay minerals, quartz, feldspars and calcite. Low-absorbing features (dark) represent low-density materials, including pore, fractures and kerogen (Figs. 5 ▶, 6 ▶ and 7 ▶). However, it is a non-trivial task to accurately determine appropriate binary threshold values in a multiphase material as in shale because the gray-level distribution is continuous, lacking clearly defined peaks or valleys in the histogram (Fig. 4 ▶). In addition, intermediate gray shades are very difficult to segment owing to low contrast and blurred boundaries from small grain sizes. Automatic thresholding algorithms such as histogram shape-based, clustering-based, and mean or mode value-based thresholding (Sezgin & Sankur, 2004 ▶) are thus not applicable to our datasets. The choice of threshold interval was therefore manually chosen based on visual inspection of low-density features and pyrite. For instance, a threshold level of low-density features in B1 collected from APS was set between the minimum grayscale of the pixels belonging to the low-density features (−10.61) and their maximum gray value (2.46) (Fig. 5*c* ▶). This threshold range sufficiently distinguishes low-density features (foreground) from shale matrix (background) and allows the objects to be further analyzed. Fig. 5(*d*) ▶ illustrates the thresholding boundary of pyrite with grayscale values between 13.52 and 42.64. After obtaining a binary image, overlapping objects were separated using the ‘Watershed’ tool and the 3D surface constructed *via* the ‘Surface Generation and Surface View’ tool in *Avizo*. The volume as well as length and width of an individual object were also determined from the ‘I-Analyze’ tool. Other datasets were quantified under the same approach. For sample N1, the VOI was chosen at 150 × 180 × 50 µm for 3D segmentation (Fig. 7 ▶). Based on these considerations the choices of threshold were selected for pyrite and low-density features and are summarized, together with corresponding volume percentages, in Table 2 ▶.

## 4. Results   

Raw projection images of sample N1 collected at each facility are quite distinctive, particularly those from the ALS and the SLS [Figs. 2(*a*) and 2(*c*) ▶] which contain several bright horizontal streaks. These stripe patterns are caused by X-ray beam inhomogeneities owing to reflections on the multilayer composition of a monochromator mirror (Table 1 ▶). The area without the sample on the ALS image is fuzzy owing to background noise. The X-ray beam fluctuation and background can be corrected to some extent with the bright- and dark-field images. Fig. 2 ▶ also shows that the cylinder axis of N1 was positioned differently and slightly inclined at each facility.

Reconstructed slices of sample N1 [Figs. 3(*a*)–3(*c*) ▶] and B1 [Figs. 3(*d*)–3(*f*) ▶] perpendicular to the cylinder axis (in the *XY*-plane) are displayed on the same brightness and contrast scale. For each facility a similar section was identified based on unique characteristic features. Low-density features (dark areas) indicate pores (small circular spots), fractures (large irregular penny-shaped) and kerogen. Fine details of pore and fracture networks can be clearly illustrated by the data collected from the APS and the SLS whereas the data from the ALS might represent only coarser features (Figs. 3 ▶, 6 ▶ and 7 ▶). Calcite in sample B1 is fairly coarse-grained and can be segmented (not shown). Other intermediate-absorbing materials in the matrix such as clays, quartz and feldspars are much more difficult to distinguish from each other owing to low contrast. Partial volume blurring was observed in all datasets, but most prominent in the data from the ALS. Despite performing the same level of ring removal, the reconstructed slices from the ALS [Figs. 3(*a*) and 3(*d*) ▶] and APS [Figs. 3(*b*) and 3(*e*) ▶] still have concentric ring artifacts in the images while the data from the SLS contain none [Figs. 3(*c*) and 3(*f*) ▶].

Two main elements were segmented to illustrate the 3D internal microstructure (Figs. 6 ▶ and 7 ▶) and to calculate volume fractions (Table 2 ▶) and aspect ratio. The resolution of the system is of the order of two pixels (*e.g.* for the SLS, 0.74 µm × 2 = 1.44 µm); therefore, any feature smaller than 3 µm^3^ [*i.e.* (1.44 µm)^3^ = 2.99 µm^3^] was excluded from calculations owing to the limit of the resolution. In both samples pyrite is generally spherical, organized into small clusters, and dispersed throughout the sample [Figs. 6(*b*), 6(*d*) ▶, 7(*a*) and 7(*c*) ▶]. In sample N1, the average volume of pyrite was estimated at 5.6%, with a slight variation between data obtained from the ALS (5.0%), APS (5.7%) and SLS (6.1%).

Pyrite is much less abundant in sample B1, with an average volume of 2.0%. Minor variation was also observed between data collected from different facilities (ALS 1.8%, APS 2.0% and SLS 2.3%). In contrast to pyrite, the shape of low-density features, including pores, fractures and kerogen, is mostly flat and penny-shaped like Figs. 6(*e*) ▶ and 7(*d*) ▶. Small low-density features (<10 µm^3^) are scattered throughout the sample while the large ones are aligned roughly parallel to the bedding plane (Fig. 6*e* ▶). Some kerogen has irregular shape but is oriented horizontally (Fig. 7*d* ▶). The average volume fraction of low-density features in sample N1 (6.3%) is higher than in sample B1 (4.5%). In addition, the volume fractions of low-density features and pyrite in both samples extracted from the APS and SLS data are more closely consistent (Table 2 ▶). Segmentation from the ALS data again yields a lowest volume estimation in both phases and samples (Table 2 ▶). The standard deviation (SD) and relative standard deviation [%RSD = (SD/Average) × 100%] were then calculated in order to compare the precision of different measurements of varying magnitudes (Table 2 ▶). The %RSD of phase volumes are quite comparable, particularly those of low-density features (9.91%) and pyrite (9.94%) in sample N1, as well as that of low-density features in sample B1 (8.91%). The similarity of %RSD suggests that these measurements have more or less the same precision. Segmentation of pyrite in sample B1 has the highest %RSD (12.38%) probably due to its lowest average-volume magnitude.

**Figure 8:**
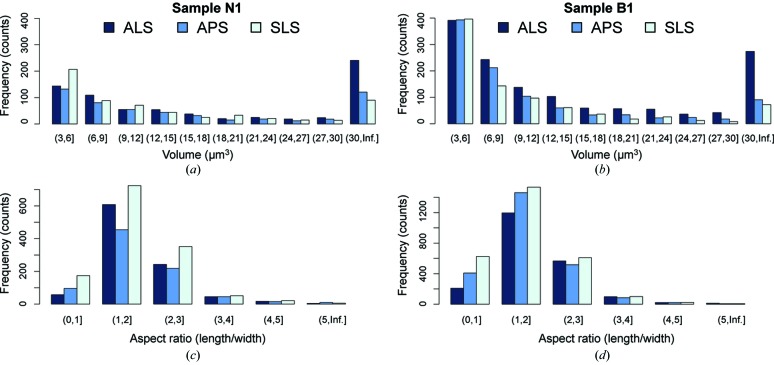
Histogram plots depict (*a*)–(*b*) the volume distribution of low-density features and (*c*)–(*d*) their aspect ratios (length/width) in samples N1 and B1, respectively.

The 3D segmentation images [Figs. 6(*e*) ▶ and 7(*d*) ▶] illustrate the shape and alignment of low-density features in both samples. The aspect ratio (length/width) of low-density features was also quantified, mostly ranging between 1 and 3 [Fig. 8(*c*) and 8(*d*) ▶]. This suggests that their shape is mainly elongated, oblate or penny-shaped. The volume distribution shows that the majority of low-density features are between 3 and 6 µm^3^ [Figs. 8(*a*)–8(*b*) ▶]. The abundance of these small and scattered features is clearly visible in Figs. 6(*e*) and 6(*d*) ▶. Some large low-density features (kerogen) (>100 µm^3^) were also identified and aligned more or less parallel to the bedding plane [Figs. 7(*b*)–7(*d*) ▶].

## 5. Discussion   

Third-generation synchrotrons provide high brilliance and intensity to produce high-quality SRXTM images for fine-grained shales. The data collected from each facility depict various 3D internal features of different samples and the same microstructures can be identified. Pyrite and low-density features, including pores, fractures and kerogen, are the main elements that can be clearly observed, segmented and quantified for relative abundances, volume distributions and shape identification (Figs. 6 ▶–8 ▶ ▶). As a number of studies suggest, lattice- and shape-preferred orientation of constituent phases in shales have a strong influence on elastic anisotropy and directionality of acoustic velocities (Sayers, 1994 ▶). For the application to shale seismic anisotropy, the shape distribution (aspect ratio) of low-density features is of most interest as the information can be used in anisotropic effective medium modeling for velocities (Hornby *et al.*, 1994 ▶).

The volume of low-density features in sample N1 obtained from SRXTM images [6.3 (6)%] is higher than the porosity reported in a previous study (2.5%) (Hornby, 1998 ▶) for several reasons. First, the volume of the low-density feature includes not only porosity but also fractures and kerogen. The previous porosity was derived from a mercury injection capillary pressure experiment (MICP), which measures pores at the nanometer scale as the sample is compressed under high pressure. SRXTM cannot image pores on the nanoscale and our measurement was performed at ambient pressure, at which the pore spaces were not closed up as tightly as in the MICP experiment. The total pore volume is thus inconsistent due to different experimental conditions. In addition, the results of the current study could be biased by the selected regions of interest, which were chosen because of the presence of large unique features (*e.g.* kerogen and fractures) that can be obviously identified in the three datasets. Thus the selected area is rather a small and heterogeneous region, which might not be a good representative of the overall porosity. An overestimation of porosity could also be due to a too high maximum threshold interval (Table 1 ▶). However, the volume distribution (Table 2 ▶) and aspect ratio calculations (Fig. 8 ▶) on the same selected area obtained from different facilities show fairly consistent results.

A small discrepancy of the volume fraction determined from each facility is due to several factors (Table 2 ▶). First, the selection of threshold values affects how volume proportions are determined. Although the data segmentation was performed on the same basis, it is difficult to precisely choose threshold values that identically represent the desired features in different datasets. Secondly, blurriness is present in all datasets but is most prominent in the reconstructed data of the ALS (Figs. 3 ▶, 6 ▶ and 7 ▶). The image blurring (*d*) is due to the finite size of the photon source (*D*) as described by *d* = *l*/(*L*/*D*), where *l* is the sample-to-detector distance and *L* is the sample-to-source distance (Schillinger *et al.*, 2000 ▶). From this equation it is obvious that a large photon source size and long sample-to-detector distance in the SRXTM system can lead to a high degree of blurriness. This is evident as the photon source size and sample-to-detector distance of the ALS (15 mm) are significantly larger than those of the APS (6 mm) and SLS (5 mm) (Table 1 ▶), causing more blurring and phase contrast in the reconstructed images. Phase contrast affects the spatial resolution as it is generated by a phase shift or interference phenomena of Fresnel fringes. The resolution limit of edge-enhanced systems is approximated by (λ*l*)^1/2^, where λ is the wavelength and *l* is the sample-to-detector distance. From this equation it can be inferred that a smaller sample-to-detector distance leads to a better spatial resolution. Since this distance varies greatly amongst facilities (5–15 mm), the effect of phase contrast on the images would also be significantly different (Figs. 3 ▶ and 4 ▶). Also, phase contrast is more pronounced when X-rays pass through a large amount of phase boundaries, such as those of low-density features. These factors thus affect the spatial resolution and the quantification of interested features as a result. Artifacts are another factor that affect the data quality and volume calculation. Concentric ring artifacts were observed in the reconstructed data of the ALS and APS [Figs. 3(*a*), 3(*b*), 3(*d*), 3(*e*) ▶, 7(*a*) and 7(*b*) ▶] owing to photon interactions, X-ray intensity fluctuations, sensitivity and defective pixels in the detector and/or scintillator (Vidal *et al.*, 2005 ▶). Other artifacts can also be transferred from the mathematical reconstruction algorithm, but this factor is less likely to create more artifacts between datasets here. Beam-hardening artifacts are typically observed in data collected from conventional X-ray sources (Baruchel *et al.*, 2000 ▶) but not from SRXTM images. The differential absorption of the polychromatic X-ray beam by the sample causes sample borders in the reconstructed slices to be brighter and yields a misleading calculation of the linear absorption coefficients.

Poor spatial resolution in SRXTM images can also be improved. The spatial resolution (*R*) can be described by *R* = [(*p*/NA)^2^ + (*q* *x*NA)^2^]^1/2^, where NA is the numerical aperture, *x* is the scintillator thickness, and *p* and *q* are constants (Stampanoni *et al.*, 2002 ▶). From this equation the numerical aperture and the scintillator thickness are the main factors that determine the spatial resolution. For each scintillator thickness, an optimal NA is necessary for achieving high spatial resolution. Besides, the scintillator material can affect the spatial resolution. Single-crystal lutetium aluminium garnet doped with cerium (LuAG:Ce) is used at the APS and SLS whereas single-crystal caesium iodide doped with thallium (CsI:Tl) (Table 1 ▶) is employed at the ALS. The LuAG:Ce scintillator is more efficient and able to achieve higher resolution that the CsI:Tl scintillator. The set-up of BL8.3.2 at the ALS is optimized for lower magnification, *e.g.* 5× and 2× objective lens. With increasing magnification (*e.g.* 10×), the depth of focus of visible-light optics is decreasing, thus a thinner scintillator and appropriate NA are necessary for improving spatial resolution at the ALS.

Limitations on spatial resolution and different sources of artifacts as well as blurriness introduce challenges into visualization and quantitative extraction of constituent phases in shales with a wide range of grain sizes and phases of different absorption characteristics. SRXTM methods may be complemented with nanoscale approaches such as focused-ion-beam scanning electron microscopy (Keller *et al.*, 2011 ▶; Bera *et al.*, 2011 ▶), transmission electron microscopy (Kanitpanyacharoen *et al.*, 2011 ▶) and X-ray nanotomography (Nelson *et al.*, 2011 ▶; Grew *et al.*, 2010 ▶). Overall, this round-robin experiment of SRXTM documented that all three beamlines produce similar results with microstructural resolution of approximately 2 µm. The 3D images of low-density features and pyrite crystals, as well as derived morphological information such as volume fractions, size distributions and aspect ratios, are consistent among the facilities, suggesting that this methodology is robust and ready to be applied to similar samples in the future.

## 6. Conclusions   

SRXTM non-destructively provides visualization and characterization of microstructural features of shales. Shales are challenging samples because many microstructural features are on the micrometer scale, at the limit of the resolution. The round-robin project has helped us to identify critical parameters in instrument capabilities as well as data processing and to make corresponding improvements. The samples used in this study are available to other laboratories for comparison purposes. State-of-the-art SRXTM proved to be a valuable tool to address various open questions in the geological field.

## Figures and Tables

**Table 1:** Specifications of equipment and acquisition parameters at each synchrotron facility

	Advanced Light Source	Advanced Photon Source	Swiss Light Source
Beamline	8.3.2	2-BM-B	TOMCAT
X-ray source	Super bend magnet 4.4 T	Bending magnet 0.6 T	Super bend magnet 2.9 T
	Ring current 500 mA	Ring current 100 mA	Ring current 400 mA
	Ring energy 1.9 GeV	Ring energy 7 GeV	Ring energy 2.4 GeV
Photon source size	220 µm × 25 µm	92 µm × 26 µm	53 µm × 16 µm
Beam size at sample	40 mm × 4.6 mm	25 mm × 4 mm	40 mm × 4 mm
Beam flux	∼10^2^ *hv* s^−1^ µm^−2^	∼10^2^ *hv* s^−1^ µm^−2^	6.8 × 10^5^ photons s^−1^ µm^−2^
Monochromator type	Multilayer (W/B_4_C), wide bandpass ∼1%	Double-crystal multilayer, unfocused	Double-crystal multilayer, bandwidth 2–3%
Monochromator-to-source distance	14 m	27.4 m	7 m
Sample-to-source distance	20 m	50 m	25 m
Sample-to-detector distance	15 mm	6 mm	5 mm
Scintillator type	Single-crystal caesium iodide doped with thallium (CsI:Tl) (λ ≃ 550 nm)	Single-crystal lutetium aluminium garnet doped with cerium (LuAG:Ce) (λ ≃ 535 nm)	Single-crystal lutetium aluminium garnet doped with cerium (LuAG:Ce) (λ ≃ 535 nm)
Scintillator thickness	35 µm	50 µm	20 µm
Detector type	CCD: Cooke PCO 4000	CCD: CoolSNAP K4 from Photometrics	CCD: PCO2000
Detector resolution	4008 × 2672 (14-bit)	2048 × 2048 (14-bit)	2048 × 2048 (14-bit)
Objective len	Mitutoyo 10× (NA = 0.27)	Zeiss Axioplan 10× (NA = 0.20)	Olympus Uplapo 10× (NA = 0.40)
Pixel size (µm)	0.88 × 0.88	0.72 × 0.72	0.74 × 0.74
Exposure time (ms)	1500	200	200
Angular increment (°)	0.120	0.120	0.120
No. of projections	1500	1500	1500
No. of bright-field images	12	20	200
No. of dark-field images	5	20	20

**Table 2:** Selected absorption threshold values and volume fractions of pyrite and low-density features in samples N1 and B1 The average, standard deviation (SD) and relative standard deviation (%RSD) of phase volumes are also shown. Note that %RSD is (100 × SD/Average).

		Grayscale thresholds (low-density features)		Low-density	Grayscale thresholds (pyrite)		Pyrite
Sample	Source	Min	Max	features (vol%)	Min	Max	(vol%)
N1	ALS	−30.37	3.33	5.6	18.38	72.55	5.0
	APS	−12.58	3.52	6.5	11.67	54.32	5.7
	SLS	−18.25	4.38	6.8	17.84	86.37	6.1
Average				6.3			5.6
SD				0.62			0.56
%RSD				9.91%			9.94%

B1	ALS	−23.67	4.49	4.1	24.00	68.97	1.8
	APS	−10.61	2.46	4.6	13.52	42.64	2.0
	SLS	−15.16	4.00	4.9	21.32	65.86	2.3
Average				4.5			2.0
SD				0.40			0.25
%RSD				8.91%			12.38%
